# Application of poly(vinylphosphonic acid) modified poly(amidoxime) in uptake of uranium from seawater

**DOI:** 10.1039/d1ra09118b

**Published:** 2022-01-31

**Authors:** Yangchun He, Guangshun Hou, Xirui Lu, Pengpeng Chang, Dadong Shao

**Affiliations:** School of Environmental and Biological Engineering, Nanjing University of Science and Technology Nanjing 210094 P. R. China shaodadong@126.com; Institute of Resources and Environment, Henan Polytechnic University Jiaozuo 454000 P. R. China; Fundamental Science on Nuclear Wastes and Environmental Safety Laboratory, Southwest University of Science and Technology Mianyang 621010 P. R. China; CNNP Jiangsu Nuclear Power Co. Ltd. Lianyungang 222042 P. R. China

## Abstract

To enhance the anti-biofouling properties and adsorption capability of poly(amidoxime) (PAO), vinylphosphonic acid (VPA, CH_2_=CH-PO_3_H_2_) was polymerized on poly(acrylonitrile) (PAN) surface by plasma technique, followed by amidoximation treatment to convert the cyano group (–C≡N) into an amidoxime group (AO, –C(NH_2_)=N–OH). The obtained poly(vinylphosphonic acid)/PAO (PVPA/PAO) was used as an adsorbent in the uptake of U(vi) from seawater. The effect of environmental conditions on the anti-biofouling property and adsorption capability of PVPA/PAO for U(vi) were studied. Results show that the modified PVPA enhances the anti-biofouling properties and adsorption capability of PAO for U(vi). The adsorption process is well described by the pseudo-second-order kinetic model and reached equilibrium in 24 h. Adsorption isotherms of U(vi) on PVPA/PAO can be well fitted by the Langmuir model, and the maximum adsorption capability was calculated to be 145 mg g^−1^ at pH 8.2 and 298 K. Experimental results highlight the application of PVPA/PAO in the extraction of U(vi) from seawater.

## Abstract 2

Our modified poly(vinylphosphonic acid) (PVPA) enhances the anti-biofouling properties and adsorption capability of poly(amidoxime) (PAO); the PVPA/PAO presents exceptional ability in the selective uptake of U(vi) from seawater.

## Graphical abstract

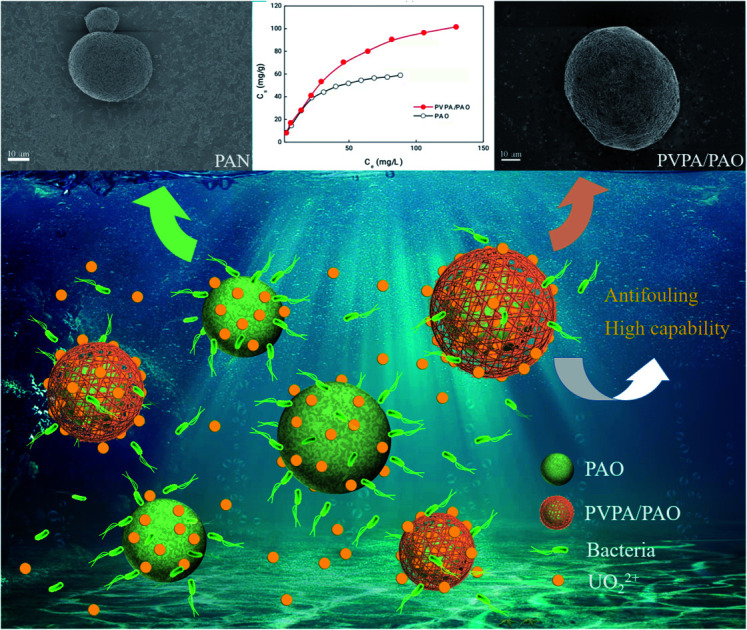

## Introduction

Uranium is very important in the nuclear industry, but uranium ores could be exhausted in a few decades. Researchers have been seeking new uranium sources for half a century. Uranium (U(vi)) in seawater is the only alternative source on Earth, and could meet the growing demand for thousands of years.^1–5^ However, the task of extracting U(vi) from seawater faces many practical problems, such as its very low concentration, unpredictable ocean weather, and destructive marine biofouling *etc.*

The development of specific materials with excellent anti-biofouling properties, high adsorption capability and good reusability are critical for U(vi) recovery. PAO based materials are widely applied in U(vi) separation from seawater because of the strong coordination capability between the –C(NH_2_)=N–OH group and U(vi).^6–8^ However, the adsorption capability of PAO based materials is strongly restrained by its poor anti-biofouling properties. Marine organisms are the foundation of the marine ecosystem, and marine biofouling refers to the undesired accumulation and growth of marine organisms on material surfaces when immersed in seawater.^9,10^ A long operation process is necessary to achieve the target enrichment of U(vi) from seawater, and unwanted marine biofouling is inevitable. It can significantly decrease the stability, adsorption capability, and reusability of an adsorbent.^11,12^ Therefore, effectively solving the biofouling problem is crucial for PAO based materials when used in U(vi) extraction.

It is strategically important to design economical materials with sound anti-biofouling capability and good affinity towards U(vi) in seawater.^11,12^ Researchers^12,13^ have found that the modification of hydrophilic/acidic groups is a feasible method to enhance the hydrophilic, anti-biofouling properties and adsorption capability of PAO based materials. Phosphorylated reagents are widely used as extraction reagents for U(vi) separation^7,12,14,15^ and are used as disinfectants^16^ due to their high affinity for U(vi) and excellent broad-spectrum anti-microbial properties, respectively. Surface modification with phosphorylated reagents is an attractive method to improve the anti-biofouling property and adsorption capability of PAO based materials.^17,18^ Among the reported phosphorylated organic monomers, VPA is a simple structure, has low toxicity, and is an industrially available monomer.^19–21^ The typical radical polymerization method of PVPA usually suffers from impurities,^19,22,23^ very slow reaction rate,^23^ underutilization of reagents,^24^ and serious chemical pollutants.

To enhance the anti-biofouling properties and adsorption capability of PAO, PVPA was modified on the PAO surface. Briefly, VPA was polymerized on the PAN surface by plasma technique, followed by amidoximation treatment. To evaluate the anti-biofouling properties and adsorption capability of PVPA/PAO, the well-known marine microorganism *V. alginolyticus*^11,25^ was selected as a representative of marine microorganisms. The effects of environmental conditions were studied. We found that the modified PVPA enhances the anti-biofouling properties and adsorption capability of PAO, and PVPA/PAO has excellent properties in U(vi) recovery.

## Results and discussion

### Characterization

**Fig. 1:**
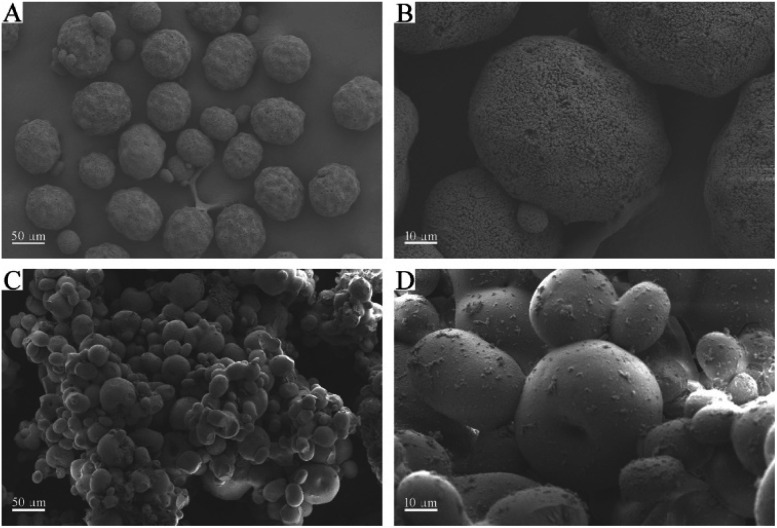
SEM images of PAN (A, B) and PVPA/PAO (C, D).

Surface topology provides direct information about the formation of PVPA/PAO. The surface morphologies of PAN are spherical in shape with wrinkles and numerous superficial holes (Fig. 1A and B). After modification with PVPA and amidoximation treatment, the surface morphologies of PVPA/PAO (Fig. 1C and D) are still spherical in shape, but the holes can hardly be seen. Instead, due to the cohesive force generated from PVPA, smooth and agglomerated surfaces of PVPA/PAO are clearly recognized. All this indicates the successful formation of PVPA/PAO.

**Fig. 2:**
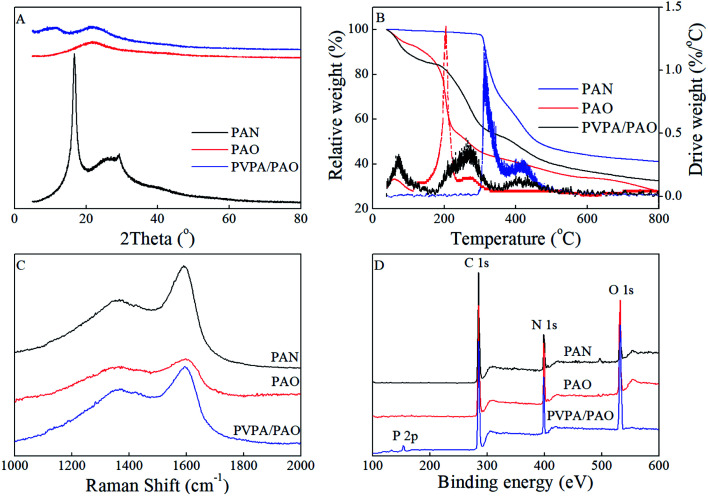
XRD patterns (A), TGA curves (B), Raman spectra (C) and XPS survey spectra (D) of PAN, PAO and PVPA/PAO.

To evaluate the effect of plasma and amidoximation treatment on the PAN framework, PAN and PVPA/PAO are characterized by XRD. The XRD pattern (Fig. 2A) of PAN shows typical peaks related to PAN at 2*θ* = 16.8° and 29.3°, which cannot be detected in the XRD patterns of PAO and PVPA/PAO. The latter shows a typical peak at 2*θ* 21.8° related to PAO. PVPA/PAO also shows a new peak at 2*θ* = 11.2° related to PVPA, which confirms the successful synthesis of PVPA/PAO.

PVPA/PAO was also characterized by TGA curves (Fig. 2B) to evaluate its thermal stability. Since AO and VPA are hydrophilic functional groups, the weight loss of moisture (before 115 °C) in PAO and PVPA/PAO was ∼7.1% and ∼12.1%, respectively. The decomposition temperatures of PAN and PAO are ∼291–492 and ∼151–328 °C, respectively. The TGA curve of PVPA/PAO depicts the typical decomposition of PVPA and PAO. The ∼31.0% weight loss at ∼151–328 °C is related to the pyrolysis of PAO and the dehydration of PVPA.^24,26,27^ The new ∼12.8% weight loss at ∼363–520 °C as compared to PAO, could be due to the pyrolysis of PVPA. PAO and PVPA/PAO lose ∼27.9% and ∼32.5% at 800 °C, respectively. Combined with the fact that PVPA typically loses ∼40% at 800 °C when it degrades in nitrogen,^27^ the PVPA weight percent in dry PVPA/PAO and PVPA/PAO mass ratio were roughly estimated to be 38% and 0.62 : 1, respectively. This result reveals the effective modification of PVPA using the plasma technique.

The disorder carbon structure (D band) materials resonate with adjacent atoms and then affect the graphite carbon structure (G band) materials,^28^ which can be revealed by Raman spectroscopy. As depicted in Fig. 2C, PAN, PAO and PVPA/PAO show typical Raman peaks at ∼1367 and ∼1591–1598 cm^−1^, which relate to the D band and G band, respectively. The G bands of PAO and PVPA/PAO are shifted to ∼1598 and ∼1594 cm^−1^ as compared to that of PAN at ∼1591 cm^−1^. The graphitic degrees were roughly evaluated by the peak intensity ratio of the G and D bands (*I*_D_/*I*_G_). The *I*_D_/*I*_G_ values are 0.67, 0.72, and 0.78 for PAN, PAO and PVPA/PAO, respectively. Raman results indicate that amidoximation treatment and PVPA modification can decrease the graphitic degree of PVPA/PAO.

**Fig. 3:**
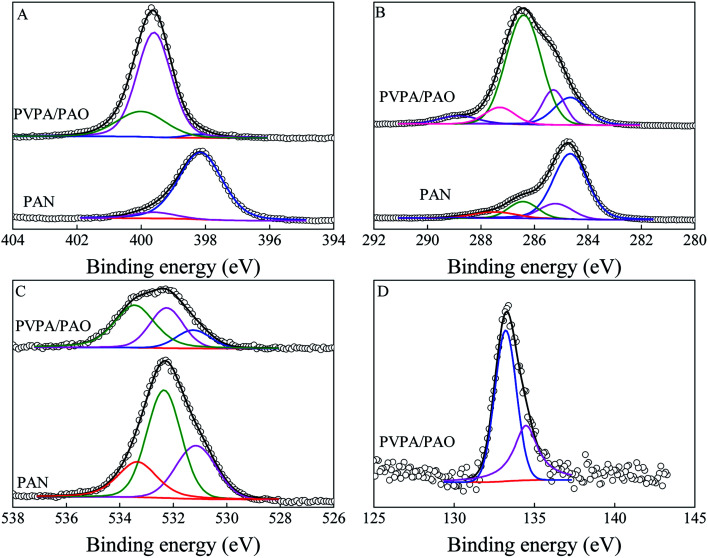
XPS N 1s (A), XPS C 1s (B), XPS O 1s (C), and P 2p (D) spectra of PAN and PVPA/PAO.

XPS spectroscopy technique can be used to identify surface functional groups. The relative peak intensities of O 1s and N 1s are increased in PAO and PVPA/PAO as compared to that of PAN (Fig. 2D), indicating –C≡N groups were successfully converted into –C(NH_2_)=N–OH groups. The new peak at ∼133 eV relates to P 2p and reveals the successful synthesis of PVPA/PAO. The N 1s spectra (Fig. 3A) were resolved into three species of –C≡N, N–H, and –C=NOH (only for PVPA/PAO). The result in Table 1 indicates that most –C≡N were converted into –C=NOH. The XPS C 1s spectra (Fig. 3B) further confirm this, and can be resolved into species –C≡N, C–C, C–OH and –C=NOH (only for PVPA/PAO); C=O, –COOH and C–PO_3_H_2_ (only for PVPA/PAO). The result in Table 2 confirms that most –C≡N was converted into –C=NOH, and C–PO_3_H_2_ is an important carbon species of PVPA/PAO. The related XPS O 1s spectra (Fig. 3C) were resolved into three species (Table 3) of –COOH, C=O and –C=NOH, and –OH and –PO_3_H_2_. The decrease of –COOH and increase of –OH and –PO_3_H_2_ confirm that PVPA/PAO was synthesized successfully. The P 2p spectrum of PVPA/PAO (Fig. 3D) is deconvoluted into two species of –PO_3_H_2_ and polyphosphate (contains P–O–P bond), which were centered at 133.21 and 134.47 eV, respectively (Table 4).^28–31^

**Table 1:** Curve fitting results of XPS N 1s spectra

	Peak	BE^a^ (eV)	FWHM^b^ (eV)	%
PAN	–C≡N	398.16	1.60	93.0
	N–H	399.60	1.50	7.02
PVPA/PAO	–C≡N	398.20	0.96	1.79
	N–H	399.60	1.26	73.6
	–C=NOH	400.00	1.79	24.6

a Binding energy.

b Full width at half-maximum.

**Table 2:** Curve fitting results of XPS C 1s spectra

	Peak	BE (eV)	FWHM (eV)	%
PAN	–C≡N	284.66	1.49	66.0
	C–C	285.20	1.43	13.4
	C–OH	286.42	1.26	13.2
	C=O	287.50	1.86	7.14
	–COOH	288.90	0.77	0.31
PVPA/PAO	–C≡N	284.66	1.43	15.2
	C–C	285.29	0.98	12.2
	–C=NOH, C–OH	286.40	1.55	60.1
	C=O	287.30	1.66	4.78
	–COOH, C–PO_3_H_2_	288.83	1.32	7.75

**Table 3:** Curve fitting results of XPS O 1s spectra

	Peak	BE (eV)	FWHM (eV)	%
PAN	–COOH	531.15	1.77	27.9
	C=O	532.35	1.51	50.3
	–OH	533.35	1.73	21.8
PVPA/PAO	–COOH	531.25	1.58	15.4
	C=O, –C=NOH	532.25	1.65	34.5
	–OH, –PO_3_H_2_	533.45	1.74	50.1

**Table 4:** Curve fitting results of XPS P 2p spectrum

	Peak	BE (eV)	FWHM (eV)	%
PVPA/PAO	–PO_3_H_2_	133.21	1.58	62.3
	Polyphosphate	134.47	1.79	37.7

### Anti-biofouling properties and adsorption capability of PVPA/PAO

**Fig. 4:**
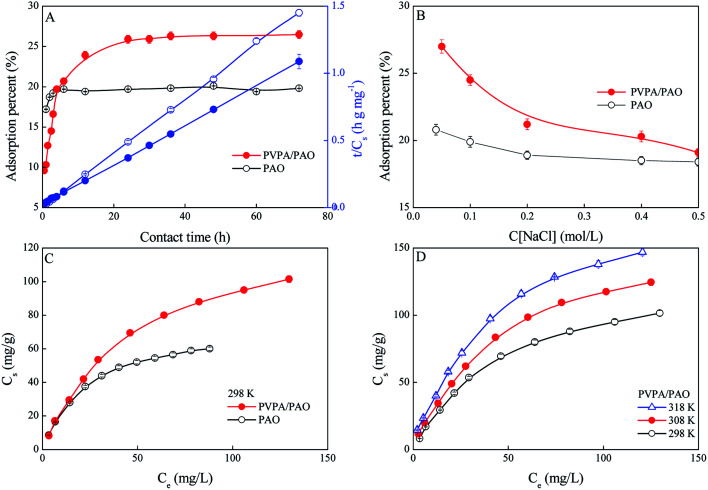
Effect of contact time (A), ionic strength (B), adsorption isotherms (C), and temperature (D) on U(vi) adsorption. mV = 0.20 g L^−1^, pH = 8.2 ± 0.1. (A): *T* = 298 ± 1 K, C[U(vi)]_initial_ = 50.0 mg L^−1^, C[NaCl] = 0.10 mol L^−1^. (B): *T* = 298 ± 1 K, contact time: 24 h, C[U(vi)]_initial_ = 50.0 mg L^−1^. (C): *T* = 298 ± 1 K, contact time: 24 h, C[NaCl] = 0.10 mol L^−1^. (D): contact time: 24 h, C[NaCl] = 0.10 mol L^−1^.

The existing forms of U(vi) are affected by solution pH, and mainly exist as U(vi)–CO_3_^2−^ species in seawater due to the weak alkalinity (pH ∼8.2) of seawater and the presence of CO_2_. The uptake of U(vi) from seawater is difficult, and fairly limited by the extremely low concentration and the decomplexation of U(vi)–CO_3_^2−^ species to free UO_2_^2+^. Thereby, an adsorbent must be soaked in seawater for a long-time during applications. To estimate the essential operating time of PVPA/PAO in applications, the influence of operation time on U(vi) extraction efficiency is evaluated. The recovery of U(vi) by PVPA/PAO rises fast with increasing reaction time upto ∼24 h, and then remains steady with further increasing reaction time (Fig. 4A). Faster kinetics need shorter operation times, which can reduce the effect of biofouling. To investigate the adsorption kinetics, the pseudo-first order kinetic models (*q*_t_ = *q*_e_ × (1 − exp(−*k*_1_*t*)), where *k*_1_ (1 h^−1^) is the adsorption rate constant, *q*_e_ (mg g^−1^) and *q*_t_ (mg g^−1^) are the equilibrium and experimental adsorption capabilities, respectively); and pseudo-second-order kinetic models (*q*_t_ = *q*_e_ × *t*/(1/(*K*′ × *q*_e_) + *t*), where *K*′ (g mg^−1^ h^−1^) is the adsorption rate constant), are used to simulate the experimental data. Based on the correlation parameters (*R*^2^) in Table 5, the adsorption of U(vi) on PVPA/PAO can be described with the pseudo-second-order kinetic model better than the pseudo-first-order kinetic model. The result indicates that U(vi) adsorption on PVPA/PAO is a chemisorption process, and the modified PVPA groups formed stable complexation with U(vi) under experimental conditions.

**Table 5:** Kinetic parameters of U(vi) adsorption

	Pseudo-first-order			Pseudo-second-order		
	*K*_1_ (1 h^−1^)	*q*_e_ (mg g^−1^)	*R* ^2^	*K*′ (g mg^−1^ h^−1^)	*q*_e_ (mg g^−1^)	*R* ^2^
PAO	2.07	48.9	0.846	6.20	49.4	0.999
PVPA/PAO	0.398	63.6	0.910	0.602	67.9	0.968

Based on the fact that NaCl is the predominant salt in seawater, the effect of NaCl on the adsorption of U(vi) on the PVPA/PAO surface was studied. As shown in Fig. 4B, the increasing NaCl concentration just slightly reduces the recovery of U(vi) by PVPA/PAO, which suggests the good selectivity of PVPA/PAO for U(vi).

To assess the adsorption capability of PVPA/PAO for U(vi), the adsorption isotherms were studied and the results are shown in Fig. 4C. The modified PVPA groups on PVPA/PAO enhance the U(vi) concentrating ability of PVPA/PAO. The experimental data are simulated by the widely used Langmuir model (*C*_s_ = *b* × *C*_s,max_ × *C*_eq_/(1 + *b* × *C*_eq_), *C*_eq_ is the equilibrium concentration of U(vi) in supernatant after centrifugation, while *C*_s,max_ (mg g^−1^) and *b* (L mg^−1^) are the maximum adsorption capability of adsorbent and the Langmuir constant, respectively) and Freundlich model (*q*_s_ = *K* × *q*_e_^1/*n*^, *K* (mg g^−1^) and 1/*n* are the constants indicative of adsorption capability and intensity, respectively). According to the *R*^2^ values in Table 6, the adsorption of U(vi) on PAO and on PVPA/PAO can be better described by the Langmuir model. The *C*_s,max_ of U(vi) on PVPA/PAO (140 mg g^−1^) is ∼1.8 times that of PAO (77.0 mg g^−1^) at pH 8.2 and 298 K.

**Table 6:** Simulated parameters of U(vi) adsorption isotherms

	*T* (K)	Langmuir model			Freundlich model		
		*C*_s,max_ (mg g^−1^)	*b* (L mg^−1^)	*R* ^2^	*K* (mg g^−1^)	1/*n*	*R* ^2^
PAO	298	77.0	0.0414	0.999	8.53	0.451	0.962
PVPA/PAO	298	140	0.0205	0.999	8.34	0.526	0.975
	308	176	0.0201	0.998	10.3	0.531	0.977
	318	203	0.0223	0.997	13.1	0.518	0.979

Adsorption isotherms of U(vi) on the PVPA/PAO surface were also performed at three different temperatures to evaluate its thermodynamic parameters. The increased reaction temperature can enhance the adsorption of U(vi) on PVPA/PAO (Fig. 4D). The thermodynamic parameters in Table 7 indicate that the recovery of U(vi) by PVPA/PAO is an endothermic and spontaneous reaction, which further confirmed the macroscopic experimental results.

**Table 7:** Thermodynamic parameters of U(vi) adsorption

*T* (K)	ln *K*_d_	Thermodynamic parameters		
		Δ*G*^0^ (kJ mol^−1^)	Δ*H*^0^ (kJ mol^−1^)	Δ*S*^0^ (J mol^−1^ K^−1^)
298	7.89	−19.6	36.6	188
308	8.41	−21.5		
318	8.81	−23.3		

**Fig. 5:**
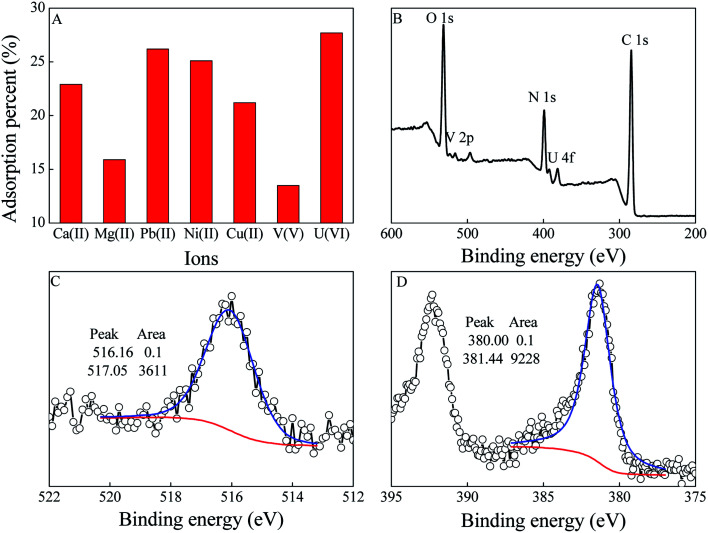
Selective adsorption of U(vi) on PVPA/PAO (A). mV = 0.20 g L^−1^, pH = 8.2 ± 0.1, *T* = 298 ± 1 K, C[ions]_initial_ = 50.0 mg L^−1^, C[NaCl] = 0.10 mol L^−1^, contact time: 24 h. XPS survey (B), V 2p (C), and U 4f (D) spectra of PVPA/PAO adsorbed U(vi) and V(v).

Ca(ii) and Mg(ii) are the predominant cations in seawater after Na(i), which can form stable ternary complexes (Ca_2_[UO_2_(CO_3_)_3_], Ca[UO_2_(CO_3_)_3_]^2−^, and Mg[UO_2_(CO_3_)_3_]^2−^) in seawater. Meanwhile, V(v) is believed to be a bigger obstacle because PAO has a stronger affinity for V(v) than U(vi). To evaluate the selectively of PVPA/PAO for U(vi), the competitive adsorption of U(vi) with Ca(ii), Mg(ii) and V(v) was measured at 50 mol L^−1^, and the results are shown in Fig. 5A. The selectivity follows the order U(vi) > Ca(ii) > Mg(ii) > V(v) in this work, which indicates high selectivity of PVPA/PAO for U(vi). The selectively of PVPA/PAO for U(vi) was further confirmed by the XPS technique. The peaks at ∼381 and ∼516 eV in the XPS survey spectrum of PVPA/PAO adsorbed U(vi) and V(v) corresponding to the V 2p and U 4f spectra (Fig. 5B). XPS V 2p and U 4f spectra were deconvoluted into V(iv) (516.16 eV) and V(v) (517.05 eV),^32^ and U(iv) (380.00 eV) and U(vi) (381.44 eV),^33^ respectively. According to Fig. 5C and D, there is no redox reaction during the adsorption of V(v) and U(vi) on PVPA/PAO surface.

**Fig. 6:**
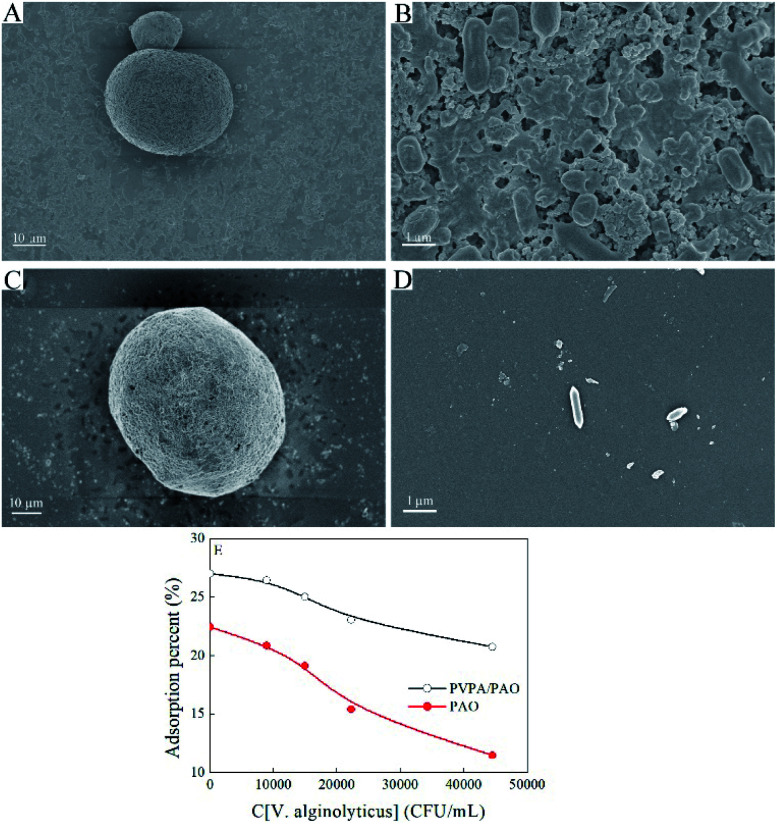
SEM images of PAO (A, B) and PVPA/PAO (C, D) after adsorbing *V. alginolyticus* and U(vi), effect of *V. alginolyticus* concentration on the uptake of U(vi) by PAO and PVPA/PAO (E). *T* = 298 ± 1 K, contact time: 24 h, C[U(vi)]_initial_ = 50.0 mg L^−1^, mV = 0.20 g L^−1^, pH = 8.2 ± 0.1, C[NaCl] = 0.10 mol L^−1^.

Biofouling in seawater is inevitable during the U(vi) extraction process. Modified PVPA significantly influences the biofouling of *V. alginolyticus* on PVPA/PAO, which was confirmed by SEM images. The enrichment of *V. alginolyticus* on PVPA/PAO (Fig. 6C and D) is much lower than that on PAO (Fig. 6A and B), which reveals the good anti-biofouling properties of PVPA/PAO. To further study the effects of biofouling on the recovery of U(vi) by PVPA/PAO, the adsorption of U(vi) on PVPA/PAO in the presence of *V. alginolyticus* under ambient conditions was evaluated. With increasing *V. alginolyticus* from 0 to 4.45 × 10^4^ CFU mL^−1^, the recovery of U(vi) by PAO and by PVPA/PAO (Fig. 6E) were decreased ∼48.9% (from ∼22.5% to ∼11.5%) and ∼23.0% (from ∼27.0% to ∼20.8%), respectively. It confirms that biofouling severely restricts the recovery of U(vi) from seawater, and PVPA/PAO has excellent anti-biofouling properties.

## Conclusions

PVPA/PAO has excellent anti-biofouling properties and high adsorption capability for U(vi). The modified PVPA on PVPA/PAO enhances its anti-biofouling property and adsorption capability for U(vi) in seawater. The enrichment of U(vi) on the PVPA/PAO surface reaches equilibrium in 24 h and follows the pseudo-second-order model. Based on the Langmuir model, the adsorption capability of PVPA/PAO for U(vi) at pH 8.2 and 298 K reaches 145 mg g^−1^.

## Experimental section

### PVPA/PAO preparation

PVPA/PAO was prepared based on plasma induced polymerization and amidoximation treatment techniques. For typical reaction conditions, 5.0 g commercial PAN powder was activated by N_2_ plasma (10 Pa, 20 W, and 940 V) for 20 min under continuous stirring, then 10 mL VPA was rapidly poured into the reactor. The polymerization of VPA on the PAN surface was performed for 24 h at room temperature under high purity N_2_ and continuous stirring. After repeatedly rinsing with ethanol, the derived material was amidoximated in 5.0 wt% NH_2_OH ethanol/water (4 : 1, v/v) at 60 °C for 3 h, and eluted with corresponding ethanol/water. The resulting PVPA/PAO was vacuum dried at 60 °C. To assess the effect of modified PVPA, PAO was synthesized by the same method.

### Characterization

To characterize the physicochemical properties of PVPA/PAO, scanning electron microscopy (SEM), Raman spectroscopy, X-ray diffraction pattern (XRD), thermogravimetric analysis (TGA), and X-ray photoelectron spectroscopy (XPS) were used. SEM images were obtained by a JSM-6320F FE-SEM (JEOL). Raman spectroscopy analysis was performed using a LabRam HR Raman spectrometer. XRD pattern was collected using a Rigaku D/max 2550 X-ray diffractometer. The TGA curve was obtained using a Shimadzu TGA-50 thermogravimetric analyser, and the heating rate and flow rate were 10 °C min^−1^ and 50 mL min^−1^ N_2_, respectively. XPS spectroscopy was obtained using a ESCALab220i-XL surface microanalysis system.

### Enrichment of *V. alginolyticus* on PAO and on PVPA/PAO

To obtain the target solutions, adsorbents (PAO and PVPA/PAO) and NaCl are pre-reacted for 24 h at first, and then deionized water and exponential growth phase *V. alginolyticus* were injected, the suspension pH was adjusted. After shaking for 24 h, the final *V. alginolyticus* concentration was analysed by dilution plate counting method.

### Effect of biofouling on U(vi) adsorption

To obtain target compositions, adsorbents (PVPA/PAO and PAO) and NaCl were pre-reacted for 24 h at first, and then deionized water, U(vi), and exponential growth phase *V. alginolyticus* were injected, and the suspension pH was adjusted. After reacting for 24 h, the suspensions were centrifuged at 9000 rpm for 30 min at 25 °C, and then filtered. Except during their specific evaluation, the reaction temperature, time, U(vi) initial concentration, mV, pH, and ionic strength are 298 ± 1 K, 24 h, 50.0 mg L^−1^, 0.20 g L^−1^, 8.2 ± 0.1, and 0.10 mol L^−1^ NaCl, respectively. The final U(vi) concentrations were analysed using Optima 2100 DV (PerkinElmer, for mg L^−1^ level) and X-Series II (Thermo Scientific, for μg L^−1^ level).

## Conflicts of interest

There are no conflicts to declare.

## Supplementary Material
